# Clinical predictive value of naïve and memory T cells in advanced NSCLC

**DOI:** 10.3389/fimmu.2022.996348

**Published:** 2022-09-02

**Authors:** Guan Zhang, Aqing Liu, Yanjie Yang, Ying Xia, Wentao Li, Yunhe Liu, Jing Zhang, Qian Cui, Dong Wang, Xu Liu, Yongtie Guo, Huayu Chen, Jianchun Yu

**Affiliations:** ^1^ Department of Oncology, First Teaching Hospital of Tianjin University of Traditional Chinese Medicine, Tianjin University of Traditional Chinese Medicine, Tianjin, China; ^2^ National Clinical Research Center for Chinese Medicine Acupuncture and Moxibustion, Tianjin, China; ^3^ Clinic Laboratory, First Teaching Hospital of Tianjin University of Traditional Chinese Medicine, Tianjin University of Traditional Chinese Medicine, Tianjin, China

**Keywords:** advanced non-small cell lung cancer, absolute counts, naïve CD4^+^ T cell, immunotherapy, prognosis

## Abstract

Currently, there is no sensitive prognostic biomarker to screen out benefit patients from the non-benefit population in advanced non-small cell lung cancer patients (aNSCLCs). The 435 aNSCLCs and 278 normal controls (NCs) were recruited. The percentages and absolute counts (AC) of circulating naïve and memory T lymphocytes of CD4^+^ and CD8^+^ T cells (Tn/Tm) were measured by flow cytometry. The percentage of CD4^+^ naïve T (Tn), CD8^+^ Tn, CD8^+^ T memory stem cell (Tscm), and CD8^+^ terminal effector T cell decreased obviously. Still, all AC of Tn/Tm of aNSCLCs was significantly lower compared to NCs. Higher AC and percentage of CD4^+^ Tn, CD8^+^ Tn, and CD4^+^ Tscm showed markedly longer median PFS in aNSCLCs. Statistics demonstrated the AC of CD4^+^ Tn (≥ 3.7 cells/μL) was an independent protective factor for PFS. The analysis of the prognosis of immunotherapy showed the higher AC and percentage of CD4^+^ Tn and CD4^+^ Tscm and higher AC of CD8^+^ Tscm had significantly longer median PFS and the AC of CD4^+^ Tn (≥ 5.5 cells/μL) was an independent protective factor for PFS. Moreover, higher AC and percentages of Tn/Tm suggested higher disease control rate and lower progressive disease rate. The AC of Tn/Tm showed more regular patterns of impairment and was more relative with the disease progression than percentages in aNSCLCs. AC had a better predictive value than percentages in Tn/Tm for PFS. Notably, the AC of CD4^+^ Tn was a potential prognostic biomarker for the PFS and efficacy of immunotherapy.

## Introduction

With the high incidence of cancer, the search for new oncology treatment strategies has become a challenging goal for researchers (1, 2). Now, the breakthrough advance is the programmed cell death protein 1 (PD-1)/programmed cell death-ligand 1 (PD-L1) monoclonal antibody, which is used in first-line immune checkpoint inhibitors (ICIs) in lung cancer (3–5). Histological detection of PD-L1 can guide treatment with anti-PD-1/PD-L1 antibodies. But PD-L1 is heterogeneous and unstable in tumors, and approximately 20% of PD-L1-negative patients still benefit from PD-1/PD-L1 antibody therapy (6). PD-L1 is not an ideal biomarker as some patients with low levels of PD-L1 expression respond to immunotherapy while others with high levels of PD-L1 expression do not, presenting a clinical challenge for patient stratification (7, 8). Some studies showed that high expression of tumor mutational burden (TMB) is associated with improved overall survival in patients with non-small-cell lung cancer (NSCLC) (9, 10). But others studies showed that high expression of TMB in patients with NSCLC is not associated with a prognosis of overall survival (11, 12). Valero C, et al. analyzed 10,233 patients (80% not treated with ICIs, 20% treated with ICIs) with 17 cancer types, before/without ICIs, or after ICIs. In non-ICIs-treated patients, higher TMB (higher percentile within cancer type) was not associated with better prognosis; in fact, in many cancer types, higher TMB was associated with poorer survival, in contrast to ICIs-treated patients, in whom higher TMB was associated with longer survival (13). These biomarkers suffer several well-known limitations, including result variability related to technical issues (14), the need for tumor biopsy tissue (15), and the lack of host immune status evaluation. The occurrence of high TMB does not always correspond to the response to ICIs (16). In an immunotherapy study for advanced NSCLC (aNSCLC), the objective response rate (ORR) for patients with high TMB and high PD-L1 expression was 62.5%, compared to 43.9% for patients with high TMB and low PD-L1 expression and 27.3% for patients with low TMB and high PD-L1 expression (17). However, PD-L1 and TMB expression did not allow assessment of the overall immune status of patients. In addition to intrinsic tumor resistance mechanisms, other reasons for this discrepancy may be directly related to the “quality” of the neoantigen, which activates naïve T cells (Tn) by Dendritic cells (DC) to generate eventual T helper cells (Th) cells and cytotoxic T lymphocytes (CTL) in response to the tumor (18). For patients with aNSCLC, immunotherapy alone or combined with chemotherapy (19) is currently the dominant mode of most clinal treatment (20). Nevertheless, recent advances showed immunotherapy combined with targeted therapy or radiotherapy did not increase the response in patients with PD-L1-positive in NSCLC (21, 22). Therefore, finding easily detectable and accurately prognostic biomarkers to guide immunotherapy is essential. Anti-PD-1/PD-L1 antibodies act by reliving immune suppression, activating immune cells to attack and destroy cancer cells (23). Several biomarkers had studied as potential candidates (24). Studies have shown promising results using absolute counts (AC) of lymphocytes in peripheral blood as a biomarker for patients with advanced NSCLC (aNSCLCs) (25, 26). In addition, CD4^+^ Th play a pivotal role in the immune system due to performing multiple functions (27, 28). The percentages of lymphocyte subsets (CD4^+^, CD8^+^ T cells, B and NK cells) could serve as predictive biomarkers of immunotherapy efficacy and progression-free survival (PFS) in NSCLC, but it did not analyze the AC of lymphocyte subsets (29). A study reported that CD8^+^PD-1^+^ to CD4^+^PD-1^+^ ratio in peripheral blood is a prognostic indicator for aNSCLCs post ICIs (30). Different memory CD4^+^ T cell subsets arise from the initial immune response of antigen-specific CD4^+^ Tn (31). Baseline CD4^+^ memory stem T cells (Tscm) in peripheral blood were associated with early screening and adjuvant diagnosis of colorectal cancer (32). The persistence of long-lived memory CD8^+^ T cells was due to their ability for clonal expansion and cytotoxicity (33–35). Our previous study found that the AC, not the percentage of lymphocyte subsets in peripheral blood of patients with NSCLC was significantly decreased compared to healthy adults. Moreover, AC is more regular than the percentage of lymphocyte subsets. There was a positive correlation between AC of CD4^+^ T cells and the PFS of patients (36). However, T lymphocyte subsets are heterogeneous populations, which can further divide into naive and memory T cells with different functions. Therefore, we hypothesized that the key to prolonging the survival of aNSCLCs with immunotherapy is the AC of T lymphocyte subsets.

Thus, we infer that the AC and percentages of circulating naïve and memory T lymphocytes of CD4^+^ and CD8^+^ T cells (Tn/Tm) could guide the clinical treatment of aNSCLCs. Hence, we explored the potential of Tn/Tm as a predictive biomarker of response to immunotherapy.

## Materials and methods

### Study design and patients

A total of 435 aNSCLCs and 278 normal controls (NCs) had recruited from the First Teaching Hospital of Tianjin University of Traditional Chinese Medicine and National Clinical Research Center for Chinese Medicine Acupuncture and Moxibustion, including 92 patients who received immunotherapy. All subjects were given informed consent under the Declaration of Helsinki. The clinical trial had approved by the Clinical Research Ethics Committee of the First Teaching Hospital of Tianjin University of Traditional Chinese Medicine (TYLL2021[K] 001). The inclusion criteria of aNSCLCs must meet: 1. had evidence of a pathological and immunohistochemical diagnosis, according to Response Evaluation Criteria in Solid Tumors (RECIST; version 1.1); 2. were the absence of other malignant tumors; 3. had complete clinical and laboratory data; 4. were at least 18 years old; 5. had absence of severe cardiac, hepatic, renal, cerebral, hematopoietic and immune system disease; 6. had an Eastern Cooperative Oncology Group performance score. Exclusion criteria included an undetermined NSCLC diagnosis or NSCLC with stage I or II, other malignant tumors, or incomplete clinical and laboratory data. All inspections of the NCs enrolled were normal, including physical examination, routine blood cell testing, liver function, renal function, and blood glucose (8, 21, 30).

**Figure 1:**
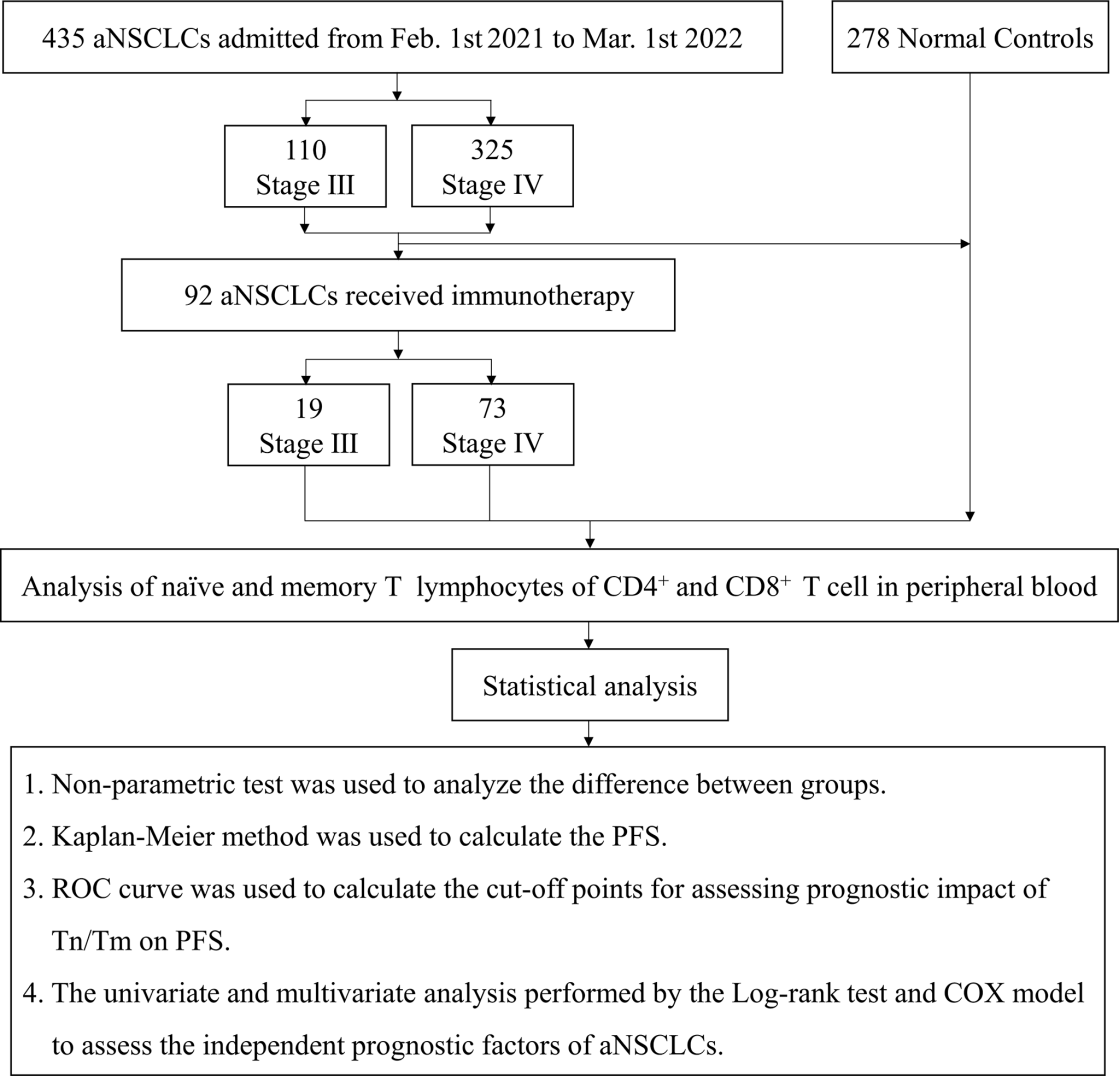
The flow chart of patient inclusion and analysis procedures in the study.

A total of 435 NSCLC patients with stages III and IV enrolled in the trial after screening between February 1, 2021, and March 1, 2022. Durvalumab was administered intravenously at a fixed dose of 1500 mg every four weeks for up to 4 sessions. Patients with aNCSLC stage III received Durvalumab combined with chemotherapy (concurrent or sequential chemotherapy). Patients with aNCSLC stage IV received Durvalumab monotherapy or Durvalumab in combination with chemotherapy (adenocarcinoma: pemetrexed in combination with carboplatin; squamous carcinoma: albumin paclitaxel in combination with carboplatin) (8).Treatment had discontinued if severe immune-related adverse events or disease progressive, or the patient or investigator decided to terminate treatment. A flow diagram of the subjects and analysis procedures for this study showed in **Figure 1**. The Tn/Tm assay had performed using flow cytometry (BD FACS Canto II: U6573380-00541, USA) with a lyse/no-wash procedure based on a single-platform technique. The known number of fluorescently labelled beads in BD Trucount tubes was the internal reference for absolute count assay.

The primary endpoint of the outcome was PFS. The second endpoints were complete response (CR), partial response (PR), stable disease (SD), and progressive disease (PD). The disease control rate (DCR) was the sum of the PR and SD rates. The differences in AC and percentages of Tn/Tm between aNSCLCs and NCs compared. In 435 aNSCLCs and 92 aNSCLCs treated with immunotherapy, PFS was compared between two groups based on the cut-off point. The baseline characteristics of patients were shown in **Table 1**. The 278 NCs included 175 males and 103 females with a median age of 67 years (range, 40-80). There were no significant differences between the aNSCLCs cohort and the NCs cohort in gender and age (P > 0.05).

**Table 1:** The baseline characteristics of aNSCLCs.

Characteristics	N = 435	(%)
**Age (years) (median, range)**	67 (39–80)	
**Sex**		
**Male**	294	67.6
**Female**	141	32.4
**Family history**		
**Yes**	103	23.7
**No**	332	76.3
**Smoking history**		
**Yes**	299	68.7
**No**	136	31.3
**Drinking history**		
**Yes**	173	39.8
**No**	262	60.2
**ECOG performance status**		
**0 or 1**	362	83.2
**≥ 2**	73	16.8
**The size of primary tumor (cm)**		
**< 2**	19	4.4
**≥ 2**	416	95.6
**Histological types**		
**Adenocarcinoma**	275	63.2
**Squamous carcinoma**	160	36.8
**Degree of differentiation**		
**Low**	390	89.7
**Moderate or High**	45	10.3
**TNM stage**		
**Ⅲ**	110	25.3
**Ⅳ**	325	74.7
**Lymph node metastasis**		
**Yes**	320	73.6
**No**	115	26.4
**Lung metastasis**		
**Yes**	184	42.3
**No**	251	57.7
**Braine metastasis**		
**Yes**	64	14.7
**No**	371	85.3
**Liver metastasis**		
**Yes**	52	12
**No**	383	88
**Bone metastasis**		
**Yes**	128	29.4
**No**	307	70.6
**Number of metastasis sites**		
**0**	49	11.3
**1**	120	27.6
**2**	183	42.1
**≥ 3**	83	19.1

### Tn/Tm assay by flow cytometry

**Figure 2:**
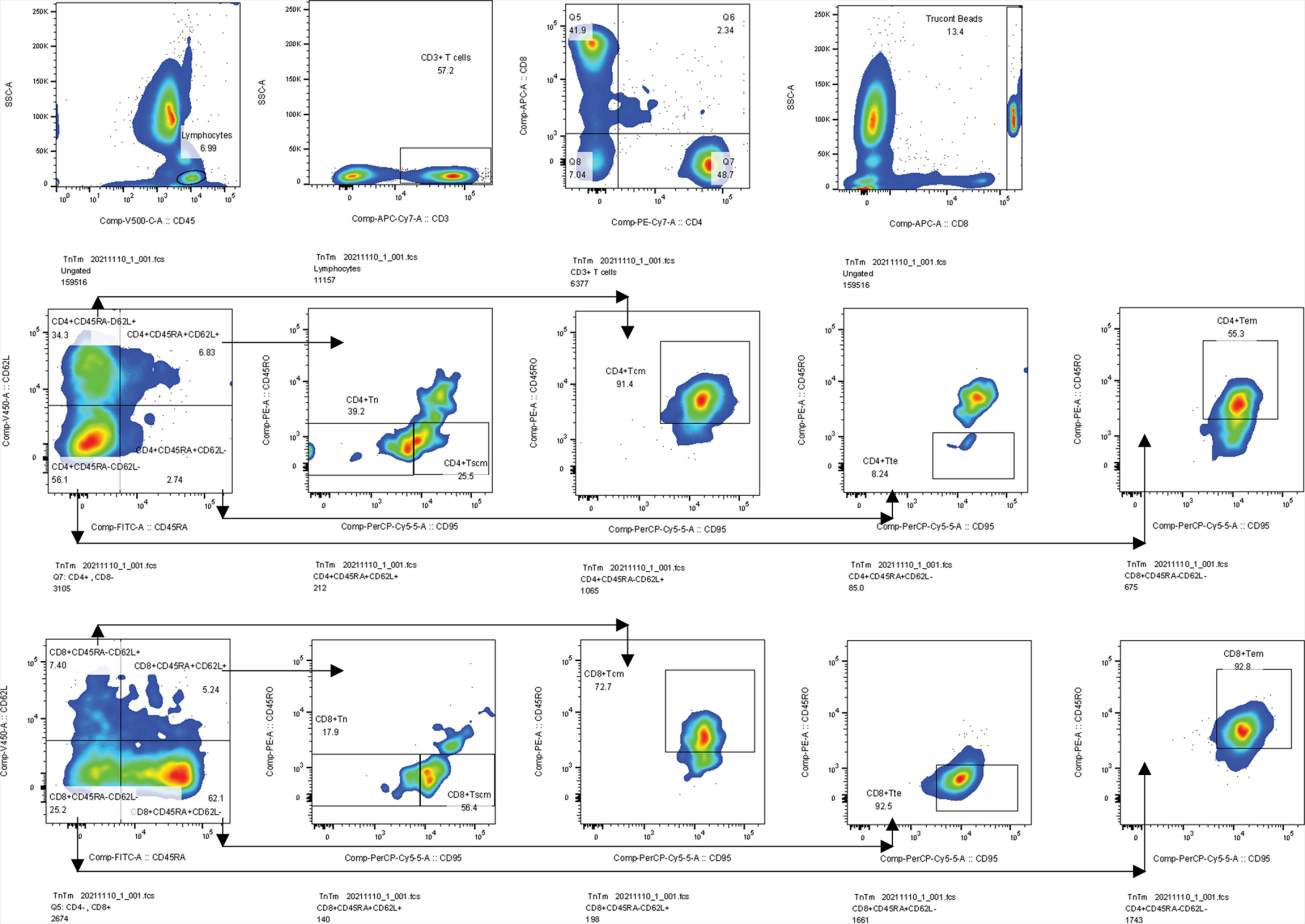
Gating strategies. Firstly, we gated lymphocytes identified by CD45 from a leukocyte, then gated CD3^+^ T cells from a lymphocyte, and gated CD4^+^ and CD8^+^ T cells from CD3^+^ T cells. Secondly, we gated Trucont Beads. Thirdly, Tn (CD95^-^CD45RO^-^) and Tscm (CD95^+^CD45RO^-^) were gated from the CD3^+^CD4^+^ CD45RA^+^CD62L^+^ T subpopulation. Tcm (CD95^+^CD45RO^+^) and Tem (CD95^+^CD45RO^+^) subpopulations were gated from CD3^+^CD4^+^CD45RA^-^CD62L^+^ and CD3^+^CD4^+^CD45RA^-^CD62L^-^ T subsets, respectively. Tte (CD95^+^CD45RO^-^) subsets were gated from the CD3^+^CD4^+^CD45RA^+^CD62L^-^ T subpopulation. The gating logic for each subset of CD3^+^CD8^+^ T cell is the same as above. Tcm, central memory T cells, Tem, effector memory T cells, Tte, terminal effector T cells.

The percentages and AC of the Tn/Tm assay were performed by the single platform technique using the ten-color flow cytometer (BD FACS Canto plus: U6573380-00541) (37). The reagents include APC-H7 Mouse Anti-Human CD3 antibody (Catalog NO: 663490), PE-Cy7 Mouse Anti-Human CD4 antibody (Catalog NO: 663493), V500-C Mouse Anti-Human CD45 antibody (Catalog NO: 662912), APC Mouse Anti-Human CD8 antibody (Catalog NO: 663524), BV421 Mouse Anti-Human CD62L antibody (Catalog NO: 563862), PerCP-Cy 5.5 Mouse Anti-Human CD95 antibody (Catalog NO: 561655), PE Mouse Anti-Human CD45RO antibody (Catalog NO: 663530), FITC Mouse Anti-Human CD45RA antibody (Catalog NO: 662840), and BD Lysing Solution (Catalog NO: 349202). The EDTA blood collecting tubes and BD Trucount tubes (Catalog NO: 340334) were also purchased from BD Biosciences, USA. The panel of phenotypic characterization of T cells was identified according to their differentiation status (**Figure 2**), such as naïve T cells (Tn) (CD45RA^+^ CD45RO^-^ CD62L^+^ CD95^-^), T memory stem cells (Tscm) (CD45RA^+^ CD45RO^-^ CD62L^+^ CD95^+^), central memory T cells (Tcm) (CD45RA^-^ CD45RO^+^ CD62L^+^ CD95^+^), effector memory T cells (Tem) (CD45RA^-^ CD45RO^+^ CD62L^-^ CD95^+^), and terminal effector T cells (Tte) (CD45RA^+^ CD45RO^-^ CD62L^-^ CD95^+^) (37).

### Sample collection, cellular staining and analyzing

Two milliliters of fresh peripheral blood were collected from all subjects using a BD Vacutainer EDTA blood collection tube. Blood samples were taken from aNSCLCs receiving immunotherapy before treatment. Tn/Tm of peripheral blood were stained and analyzed according to BD operating instruction:

1. For each sample, the corresponding BD Trucount Tube was labeled separately;
2. 5 μL each of CD3, CD4, CD8, CD45, CD62L and CD95 antibodies, and 20 μL each of CD45RA and CD45RO reagents were respectively pipetted into the bottom of labeled BD Trucount Tubes, without touching the bead pellet;
3. Reverse pipetting method was used to drew 50 μL of well-mixed, anticoagulated whole blood into the tube just above the metal retainer;
4. The tube was capped and gently rotated to mixed the antibody and the sample;
5. The mixture was placed in the dark and incubated at room temperature between 20°C and 25°C for 15 minutes;
6. 450 μL of 1 × BD Multitest IMK Kit Lysing Solution was added into the tube, covered the tube and gently shook the tube until the liquid was uniform;
7. The tube was incubated in the dark at room temperature of 20°C-25°C for 15 minutes;
8. Samples were analyzed by a ten-color flow cytometer.

A known volume of sample was stained directly in a BD Trucount Tube. The lyophilized pellet in the tube dissolves, releasing a known number of fluorescent beads. During the analysis, the absolute number of positive cells (cells per microliter) in the sample can be determined using BD FACS Canto-specific BD clinical software. This is the formula of AC of cells:


$$\text{cell absolute count }\left(\text{cells}/\text{μL}\right)\;=\frac{Acquired\;cells\;events\times Total\;Beads}{Acquired\;beads\times Voulme\;of\;sample}$$

### Statistical analysis

The comparison between groups was analyzed by non-parametric test. The median values and frequencies of the continuous and categorical variables were calculated. PFS was defined as the duration between the initiation of inclusion and the disease progression or death, whichever occurred first. PFS was calculated by Kaplan-Meier method. Patients who were still alive were reviewed at the last available follow-up. The cut-off point was calculated by ROC curve (38). Univariate and multivariate analyses were used to analyze the factors affecting disease progression. Variables with P< 0.05 in univariate analysis were entered for multivariate analysis. Log-rank test was used for univariate analysis and the proportional hazards regression model (COX model) was used for multivariate analysis. P< 0.05 were considered statistically significant. The data were analyzed by SPSS 25. 0 software and plotted by GraphPad Prism 9. 00 software.

## Results

### Comparison of Tn/Tm assay between aNSCLCs and normal controls 

**Figure 3:**
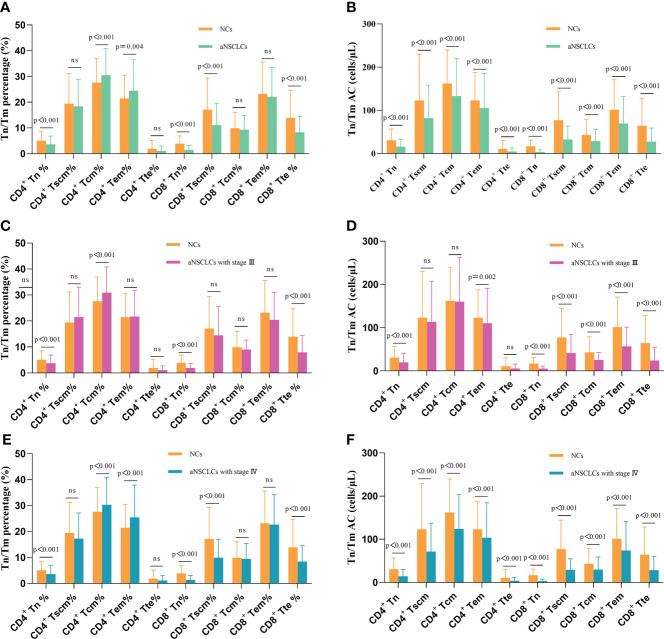
Comparison of Tn/Tm between aNSCLCs and NCs: **(A)** Comparison of percentages between aNSCLCs and NCs. **(B)** Comparison of AC between aNSCLCs and NCs. **(C)** Comparison of percentages between aNSCLCs with stage III and NCs. **(D)** Comparison of AC between aNSCLCs with stage III and NCs. **(E)** Comparison of percentages between aNSCLCs with stage IV and NCs. **(F)** Comparison of AC between aNSCLCs with stage IV and NCs. ns, no significance.

To analyze the damage status of Tn/Tm in aNSCLCs, the following comparisons were made. Compared to NCs, the percentage of CD4^+^ Tn, CD8^+^ Tn, CD8^+^ Tscm and CD8^+^ Tte in the peripheral blood of aNSCLCs decreased obviously (P< 0.001), the percentage of CD4^+^ Tcm and CD4^+^ Tem increased apparently (P< 0.05) (**Figure 3A**). Interestingly, all AC of Tn/Tm of aNSCLCs was significantly lower than NCs (P< 0.001), including CD4^+^ Tn, CD4^+^ Tscm, CD4^+^ Tcm, CD4^+^ Tem, CD4^+^ Tte, CD8^+^ Tn, CD8^+^ Tscm, CD8^+^ Tcm, CD8^+^ Tem and CD8^+^ Tte (**Figure 3B**). Compared to NCs, percentage of CD4^+^ Tn, CD8^+^ Tn and CD8^+^ Tte in aNSCLCs with stage III was lower (P< 0.001), and the percentage of CD4^+^ Tcm was higher (P< 0.001) (**Figure 3C**), whereas, the AC of CD4^+^ Tn, CD4^+^ Tem, CD8^+^ Tn, CD8^+^ Tscm, CD8^+^ Tcm, CD8^+^ Tem and CD8^+^ Tte cell was significantly lower (P< 0.05) (**Figure 3D**). In NSCLCs with stage IV, the percentage of CD4^+^ Tn, CD8^+^ Tn, CD8^+^ Tscm and CD8^+^ Tte was lower (P< 0.001), the percentage of CD4^+^ Tcm and CD4^+^ Tem was higher (P< 0.001) (**Figure 3E**), however, the AC of ten Tn/Tm subsets was significantly lower than NCs (P< 0.001) (**Figure 3F**).

The above results suggested that AC showed a more regular pattern of impairment and was more relative to the progress of the tumor than percentages of Tn/Tm in aNSCLCs. Besides, the frequencies of CD4^+^ Tn and CD8^+^ Tn decreased obviously and were associated with the development of cancer (**Table 2**).

**Table 2:** Cells impairment and prognostic cells in aNSCLCs.

Tn/Tm	Compared with the NCs						Compared with the Stage Ⅲ		PFS	
	All aNSCLCs		Stage Ⅲ		Stage Ⅳ		Stage Ⅳ		%	AC
	%	AC	%	AC	%	AC	%	AC		
CD4^+^ Tn	★	★	★	★	★	★		★	★	★
CD4^+^ Tscm		★				★	★	★	★	★
CD4^+^ Tcm	★	★	★		★	★		★		
CD4^+^ Tem	★	★		★	★	★	★		★	
CD4^+^ Tte		★				★				
CD8^+^ Tn	★	★	★	★	★	★	★	★	★	★
CD8^+^ Tscm	★	★		★	★	★	★		★	
CD8^+^ Tcm		★		★		★				
CD8^+^ Tem		★		★		★		★		
CD8^+^ Tte	★	★	★	★	★	★				

%: percentage; ★: P< 0.05.

### Comparison of Tn/Tm assay between stage III and IV in aNSCLCs

**Figure 4:**
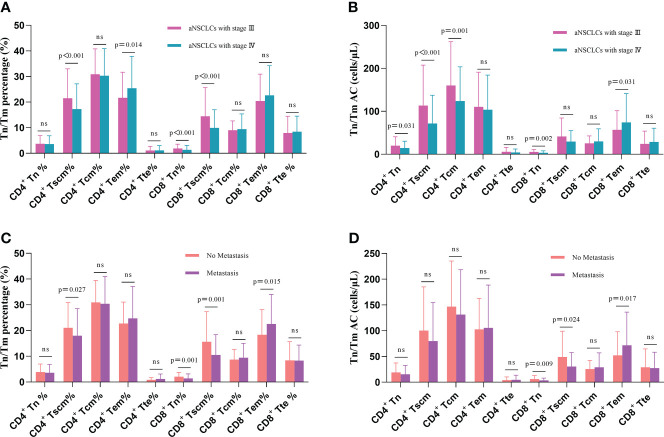
Comparison of Tn/Tm between different stages of aNSCLCs and aNSCLCs with or without metastasis: **(A)** Comparison of percentages between stages III and IV in aNSCLCs. **(B)** Comparison of AC between stages III and IV in aNSCLCs. **(C)** Comparison of percentages of aNSCLCs with or without metastasis. **(D)** Comparison of AC of aNSCLCs with or without metastasis. ns, no significance.

We further analyzed the changes in the Tn/Tm at different stages of aNSCLCs. Compared to NSCLC with stage III, the percentage of CD4^+^ Tscm, CD8^+^ Tn and CD8^+^ Tscm was lower (P< 0.001), and the percentage of CD4^+^ Tem was higher in NSCLC with stage IV (P = 0.014) (**Figure 4A**); while the AC of CD4^+^ Tn, CD4^+^ Tscm, CD4^+^ Tcm and CD8^+^ Tn was significantly lower (P< 0.05) and the AC of CD8^+^ Tem was higher (P = 0.031) (**Figure 4B**). This research further analyzed the relationship between the expression levels of Tn/Tm and metastasis (No vs Yes = 49: 386). The results showed the percentage of CD4^+^ Tscm, CD8^+^ Tn, and CD8^+^ Tscm in no metastasis was high than that in metastasis, and the frequency of CD8^+^ Tem in no metastasis was low than that in metastasis (P< 0.05) (**Figure 4C**). The AC of CD8^+^ Tn and CD8^+^ Tscm was high in no metastasis. The AC of CD8^+^ Tem was high in metastasis (P< 0.05) (**Figure 4D**).

This result suggests that the AC of Tn/Tm changes more markedly than the percentage as the disease progresses in NSCLC, and the AC of CD4^+^ Tn and CD8^+^ Tn showed a decline in change (**Table 2**). Moreover, it suggested that the percentage and AC of Tn and Tscm of CD8^+^ correlated more significantly with metastasis.

### Prognostic impact of Tn/Tm on PFS

**Figure 5:**
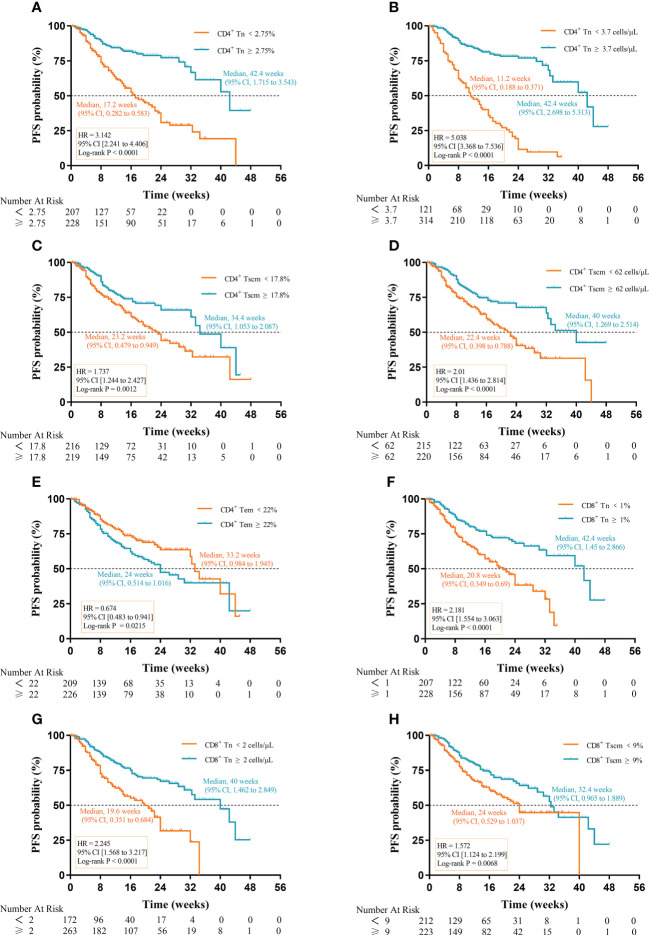
Prognostic impact of Tn/Tm on PFS of aNSCLCs: **(A)** The percentage of CD4^+^ Tn on the prognosis of PFS. **(B)** The AC of CD4^+^ Tn on the prognosis of PFS. **(C)** The percentage of CD4^+^ Tscm on the prognosis of PFS. **(D)** The AC of CD4^+^ Tscm on the prognosis of PFS. **(E)** The percentage of CD4^+^ Tem on the prognosis of PFS. **(F)** The percentage of CD8^+^ Tn on the prognosis of PFS. **(G)** The AC of CD8^+^ Tn on the prognosis of PFS. **(H)** The percentage of CD8^+^ Tscm on the prognosis of PFS.

We analyzed the relationship between AC and percentage of Tn/Tm and PFS of aNSCLCs. In **Figure 5A** (HR 3.142; 95% CI [2.241 to 4.406]; P< 0.0001), **Figure 5B** (HR 5.038; 95% CI [3.368 to 7.536]; P< 0.0001), **Figure 5F** (HR 2.181; 95% CI [1.554 to 3.063]; P< 0.0001) and **Figure 5G** (HR 2.245; 95% CI [1.568 to 3.217]; P< 0.0001), there was significantly longer median PFS for patients with higher percentage and AC of CD4^+^ Tn (42.4 weeks, 95% CI, 1.715 to 3.543; 42.4 weeks, 95% CI, 2.698 to 5.313) and CD8^+^ Tn (42.4 weeks, 95% CI, 1.145 to 2.866; 40 weeks, 95% CI, 1.462 to 2.849) compared to lower percentage and AC of CD4^+^ Tn (17.2 weeks, 95% CI, 0.282 to 0.583;11.2 weeks, 95% CI, 0.188 to 0.371) and CD8^+^ Tn (20.8 weeks, 95% CI, 0.349 to 0.69; 19.6 weeks, 95% CI, 0.351 to 0.684). High percentage of CD4^+^ Tscm was associated with better median PFS (34.4 weeks, 95% CI, 1.053 to 2.087 vs 23.2 weeks, 95% CI, 0.479 to 0.949) (HR 1.737; 95% CI [1.244 to 2.427]; P=0.0012) (**Figure 5C**). High AC of CD4^+^ Tscm was also associated with longer median PFS (40 weeks, 95% CI, 1.269 to 2.514 vs 22.4 weeks, 95% CI, 0.398 to 0.788) (HR 2.01; 95% CI [1.436 to 2.814]; P< 0.0001) (**Figure 5D**). The percentage of CD8^+^ Tscm showed a similarly change to CD4^+^ Tscm with a higher frequency and a longer median PFS (32.4 weeks, 95%, 0.965 to 1.889 vs 24 weeks, 95% CI, 0.529 to 1.037) (HR 1.572; 95% CI [1.124 to 2.199]; P = 0.0068) (**Figure 5H**). Contrarily, when the percentage of CD4^+^ Tem was low, the median PFS was higher (33.2 weeks, 95% CI, 0.984 to 1.945 vs 24 weeks, 95% CI, 0.514 to 1.016) (HR 0.674; 95% CI [0.483 to 0.941]; P = 0.0215) (**Figure 5E**). The rest of the cells, including Tcm, Tte of CD4^+^ and CD8^+^, the AC of CD8^+^ Tscm and AC of CD4^+^ Tem cells had no relationship between median PFS and cell amount (**Supplementary Figure 1**).

Notably, the difference in median PFS for AC of CD4^+^ Tn (31.2 weeks) was the most apparent, followed by percentage (25.2 weeks) of CD4^+^ Tn, percentage (21.6 weeks) of CD8^+^ Tn, and AC (20.4 weeks) of CD8^+^ Tn. The results suggested that high AC and percentage of Tn were conducive to prolonged PFS in aNSCLCs although the percentage and AC of CD4^+^ Tscm, percentage of CD4^+^ Tem, and CD8^+^ Tscm had prognostic value in PFS of aNSCLCs too (**Table 2**).

### Effect of variables on the PFS of aNSCLCs

**Figure 6:**
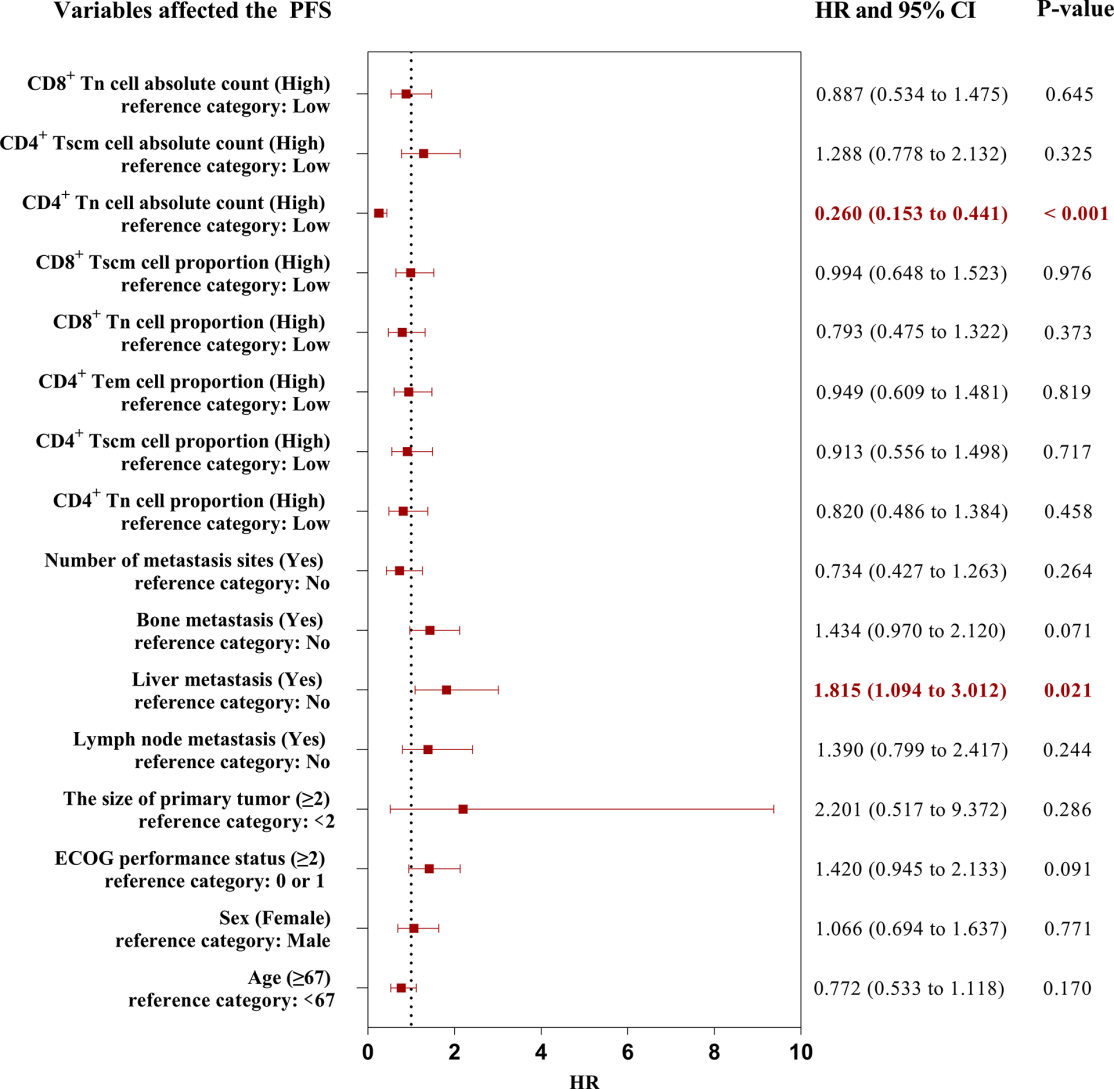
The forest plots of factors affected the PFS of aNSCLCs. HR > 1 represented variable was considered a negative factor; HR< 1 indicated variable was considered a positive factor.

Because the result had more subsets of Tn/Tm that affected the PFS of patients, it was necessary to evaluate which peripheral Tn/Tm had the most significant impact on the PFS of aNSCLCs. This study utilized univariate analysis and a Log-rank test to analyze the variables (**Table 3**). The variables of P< 0.05 were input to multivariate analysis for COX model analysis, and finally, the forest plots had drawn in **Figure 6**. The high AC of CD4^+^ Tn was an independent protective factor for PFS (HR 0.260; 95%CI [0.153 to 0.441]; P< 0.001), and liver metastasis (HR 1.815; 95%CI [1.094 to 3.012]; P = 0.021) was independent risk factor for PFS of aNSCLCs.

**Table 3:** Univariate analysis on PFS of aNSCLCs.

Univariate viable	Cutoff-point	Univariate analysis	
		P-value	HR
**Age (≥ 67 vs< 67)**	67	0.027	0.684
**Sex (Male vs Female)**		0.009	1.682
**Family history (Yes vs No)**		0.151	0.736
**Smoking history (Yes vs No)**		0.113	1.366
**Drinking history (Yes vs No)**		0.119	1.307
**ECOG performance status (≥ 2 vs< 2)**		< 0.001	1.964
**The size of primary tumor (cm) (≥2 vs< 2)**		0.033	4.57
**Histological types (Adenocarcinoma vs Squamous carcinoma)**		0.092	0.749
**Degree of differentiation (Moderate or High vs Low)**		0.955	1.015
**TNM stage (Ⅳ vs Ⅲ)**		0.6	1.109
**Lymph node metastasis (Yes vs No)**		0.036	1.531
**Lung metastasis (Yes vs No)**		0.445	1.14
**Braine metastasis (Yes vs No)**		0.061	0.554
**Liver metastasis (Yes vs No)**		< 0.001	3.024
**Bone metastasis (Yes vs No)**		0.015	1.536
**Number of metastasis sites (≥ 2 vs< 2)**		0.03	1.479
**CD4^+^ Tn cell (%) (High vs Low)**	2.75	< 0.001	0.311
**CD4^+^ Tscm cell (%) (High vs Low)**	17.8	0.002	0.575
**CD4^+^ Tcm cell (%) (High vs Low)**	30	0.087	1.346
**CD4^+^ Tem cell (%) (High vs Low)**	22	0.023	1.484
**CD4^+^ Tte cell (%) (High vs Low)**	0.85	0.186	0.785
**CD8^+^ Tn cell (%) (High vs Low)**	1	< 0.001	0.444
**CD8^+^ Tscm cell (%) (High vs Low)**	9	0.008	0.629
**CD8^+^ Tcm cell (%) (High vs Low)**	6.65	0.316	1.213
**CD8^+^ Tem cell (%) (High vs Low)**	20.6	0.058	0.721
**CD8^+^ Tte cell (%) (High vs Low)**	6.8	0.992	0.998
**CD4^+^ Tn cell (cells/μL) (High vs Low)**	3.7	< 0.001	0.189
**CD4^+^ Tscm cell (cells/μL) (High vs Low)**	62	< 0.001	0.493
**CD4^+^ Tcm cell (cells/μL) (High vs Low)**	115	0.362	0.856
**CD4^+^ Tem cell (cells/μL) (High vs Low)**	70.5	0.278	1.215
**CD4^+^ Tte cell (cells/μL) (High vs Low)**	2	0.63	0.921
**CD8^+^ Tn cell (cells/μL) (High vs Low)**	2	< 0.001	0.431
**CD8^+^ Tscm cell (cells/μL) (High vs Low)**	23	0.099	0.753
**CD8^+^ Tcm cell (cells/μL) (High vs Low)**	22	0.875	0.973
**CD8^+^ Tem cell (cells/μL) (High vs Low)**	58.5	0.297	0.834
**CD8^+^ Tte cell (cells/μL) (High vs Low)**	16	0.193	1.251

The forest plots indicated that only the AC of CD4^+^ Tn was an independent protective factor for PFS through the AC and percentage of CD4^+^ and CD8^+^ Tn, as well as the frequency and AC of CD4+ Tscm, frequency of CD4^+^ Tem, and CD8^+^ Tscm, were correlated with PFS in aNSCLCs. Thus, more attention should be paid to the AC of CD4^+^ Tn.

### Prognosis of Tn/Tm on PFS in aNSCLCs received immunotherapy

To evaluate the prognosis of Tn/Tm on PFS in aNSCLCs received immunotherapy, we analyzed the relationship between the baseline of Tn/Tm and PFS (**Table 4**).

**Table 4:** The baseline characteristics of aNSCLCs who received immunotherapy.

Characteristics	N = 92	(%)
**Age (years) (median, range)**	69 (45-80)	
**Sex**		
**Male**	64	69.6
**Female**	28	30.4
**Family history**		
**Yes**	26	28.3
**No**	66	71.7
**Smoking history**		
**Yes**	61	66.3
**No**	31	33.7
**Drinking history**		
**Yes**	35	38
**No**	57	62
**ECOG performance status**		
**0 or 1**	67	72.8
**≥ 2**	25	27.2
**The size of primary tumor (cm)**		
**< 2**	3	3.3
**≥ 2**	89	96.7
**Histological types**		
**Adenocarcinoma**	59	64.1
**Squamous**	33	35.9
**Degree of differentiation**		
**Low**	86	93.5
**Moderate or High**	6	6.5
**TNM stage**		
**Ⅲ**	19	20.7
**Ⅳ**	73	79.3
**Lymph node metastasis**		
**Yes**	65	70.7
**No**	27	29.3
**Lung metastasis**		
**Yes**	39	42.4
**No**	53	57.6
**Braine metastasis**		
**Yes**	13	14.1
**No**	79	85.9
**Liver metastasis**		
**Yes**	9	9.8
**No**	83	90.2
**Bone metastasis**		
**Yes**	26	28.3
**No**	66	71.7
**Number of metastasis sites**		
**0**	12	13
**1**	20	21.8
**2**	48	52.2
**≥ 3**	12	13
**Response evaluation**		
**Complete response (CR)**	0	0
**Partial response (PR)**	19	20.6
**Stable disease (SD)**	34	37
**Progressive disease (PD)**	39	42.4

**Figure 7:**
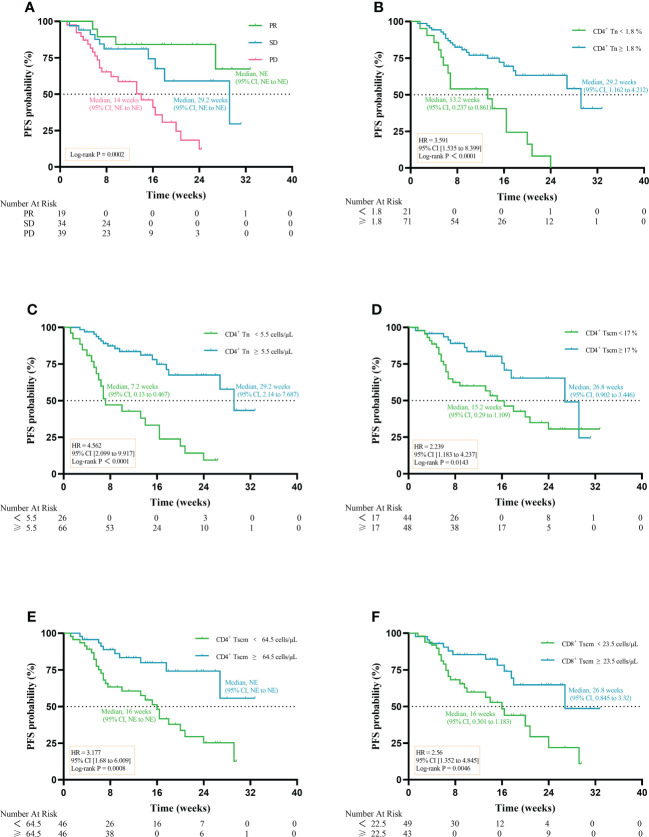
Prognostic impact of the baseline of Tn/Tm on PFS of NSCLCs: **(A)** Prognostic impact of PR, SD, and PD on PFS. **(B)** Prognostic impact in percentage of CD4^+^ Tn on PFS. **(C)** Prognostic impact in AC of CD4^+^ Tn on PFS. **(D)** Prognostic impact in percentage of CD4^+^ Tscm on PFS. **(E)** Prognostic impact in AC of CD4^+^ Tscm on PFS. **(F)** Prognostic impact in AC of CD8^+^ Tscm on PFS. NE: not estimable.

When comparing the PFS in patients with PR, SD and PD, PR had the highest median PFS (NE, 95% CI, NE to NE vs 29.2 weeks, 95% CI, NE to NE vs 14 weeks, 95% CI, NE to NE) (P = 0.0002) (**Figure 7A**). High percentage of CD4^+^ Tn was associated with longer median PFS (29.2 weeks, 95% CI, 1.162 to 4.212 vs 13.2 weeks, 95% CI, 0.237 to 0.861) (HR 3.591; 95% CI [1.535 to 8.399]; P< 0.0001) (**Figure 7B**). Patients with high AC of CD4^+^ Tn had a better median PFS compared with patients with low AC of CD4^+^ Tn (29.2 weeks, 95% CI, 2.14 to 7.687 vs 7.2 weeks, 95% CI, 0.13 to 0.467) (HR 4.562; 95% CI [2.099 to 9.917]; P< 0.0001) (**Figure 7C**). High percentage and AC of CD4^+^ Tscm in aNSCLCs had significantly longer median PFS compared with low percentage and AC of CD4^+^ Tscm in aNSCLCs (26.8 weeks, 95% CI, 0.902 to 3.446 vs 15.2 weeks, 95% CI, 0.29 to 1.109) (HR 2.239; 95% CI [1.183 to 4.237]; P = 0.0143, **Figure 7D**) (NE, 95% CI, NE to NE vs 16 weeks, 95% CI, NE to NE) (HR 3.177; 95% CI [1.68 to 6.009]; P = 0.0008, **Figure 7E**). There was a significant difference in PFS for patients with high or low CD8^+^ Tscm (26.8 weeks, 95% CI, 0.845 to 3.32 vs 16 weeks, 95% CI, 0.301 to 1.183) (HR 2.56; 95% CI [1.352 to 4.845]; P = 0.0046) (**Figure 7F**). The Tcm, Tem, Tte of CD4^+^ and CD8^+^, the percentage and AC of CD8^+^ Tn and the percentage of CD8^+^ Tscm cells had no relationship between PFS and cell amount in aNSCLCs (**Supplementary Figure 2**).

Overall, this indicated that high AC and percentage of CD4^+^ Tn and CD4^+^ Tscm were beneficial in prolonging PFS in aNSCLCs. The AC of CD8^+^ Tscm had prognostic value for PFS of aNSCLCs.

**Figure 8:**
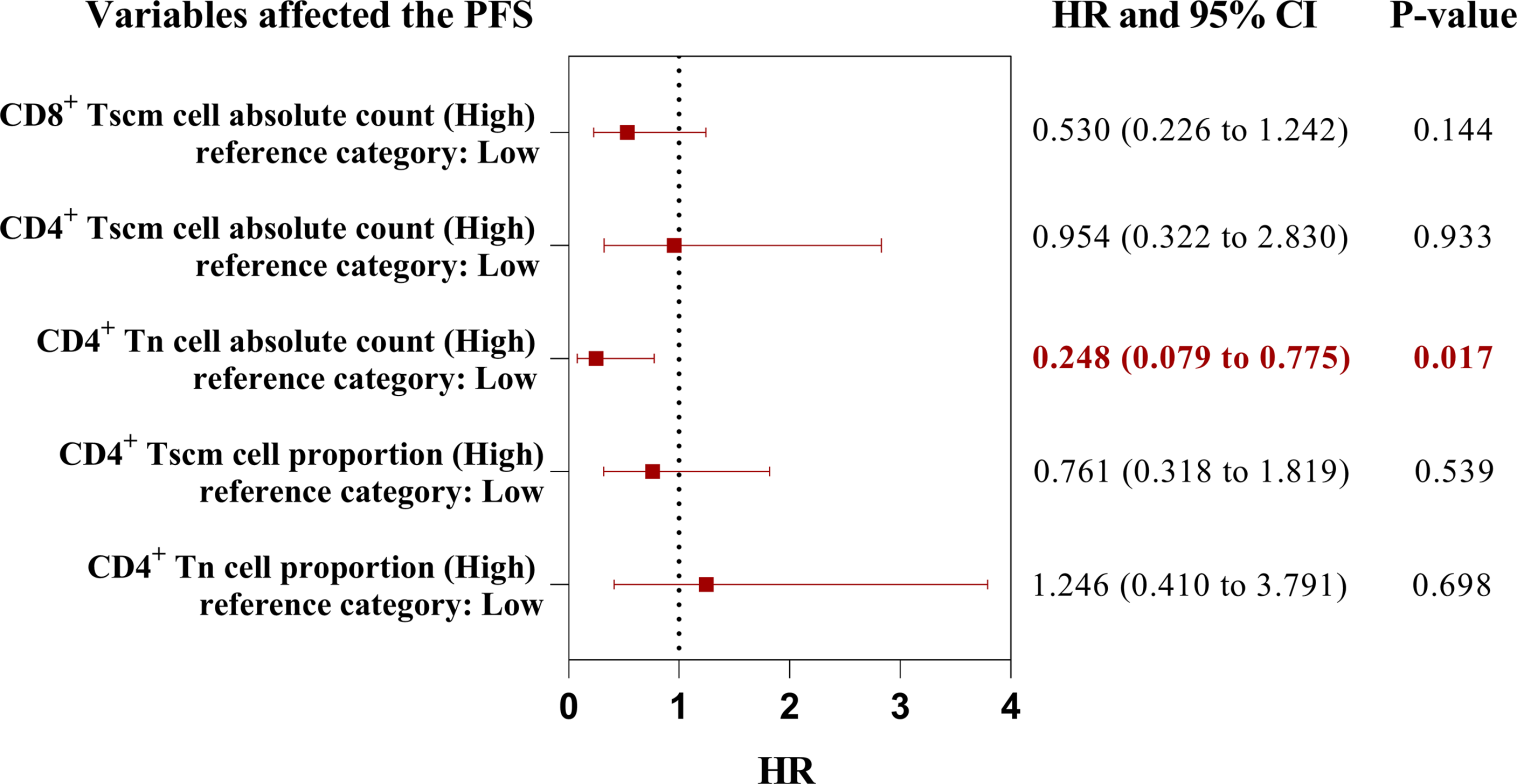
The forest plots of factors affected the PFS of aNSCLCs who received immunotherapy. HR > 1 represented variable was considered a negative factor; HR< 1 indicated variable was considered a positive factor.

The high AC of CD4^+^ Tn, CD4^+^ Tscm, and CD8^+^ Tscm and the high percentage of CD4^+^ Tn and CD4^+^ Tscm were associated with a better prognosis. We wondered which peripheral Tn/Tm subset was most relevant to the PFS of aNSCLCs who received immunotherapy. To answer this question, we conducted univariate analyses, using the Log-rank test to analyze the variables (**Table 5**). The variables with P< 0.05 entered into multivariate analysis for COX model analysis to draw forest plots. The results showed the AC of CD4^+^ Tn ≥ 5.5 cells/μL (HR 0.248; 95% CI [0.079 to 0.775]; P = 0.017) was an independent protective factor in PFS of aNSCLCs who received immunotherapy (**Figure 8**).

**Table 5:** Univariate analysis on PFS of aNSCLCs who received immunotherapy.

Univariate viable	Cutoff-point	P-value	HR
**CD4^+^ Tn cell (%) (High vs Low)**	1.8	< 0.001	0.266
**CD4^+^ Tscm cell (%) (High vs Low)**	17	0.018	0.442
**CD4^+^ Tcm cell (%) (High vs Low)**	29.85	0.542	0.819
**CD4^+^ Tem cell (%) (High vs Low)**	22.1	0.702	0.882
**CD4^+^ Tte cell (%) (High vs Low)**	0.35	0.214	0.659
**CD8^+^ Tn cell (%) (High vs Low)**	1.35	0.077	0.494
**CD8^+^ Tscm cell (%) (High vs Low)**	9.55	0.185	0.638
**CD8^+^ Tcm cell (%) (High vs Low)**	9	0.741	0.896
**CD8^+^ Tem cell (%) (High vs Low)**	21.3	0.064	0.538
**CD8^+^ Tte cell (%) (High vs Low)**	6.55	0.923	0.969
**CD4^+^ Tn cell (cells/μL) (High vs Low)**	5.5	< 0.001	0.205
**CD4^+^ Tscm cell (cells/μL) (High vs Low)**	64.5	0.002	0.313
**CD4^+^ Tcm cell (cells/μL) (High vs Low)**	116.5	0.071	0.548
**CD4^+^ Tem cell (cells/μL) (High vs Low)**	80	0.388	0.754
**CD4^+^ Tte cell (cells/μL) (High vs Low)**	2	0.14	0.616
**CD8^+^ Tn cell (cells/μL) (High vs Low)**	2	0.103	0.587
**CD8^+^ Tscm cell (cells/μL) (High vs Low)**	23.5	0.006	0.383
**CD8^+^ Tcm cell (cells/μL) (High vs Low)**	25.5	0.715	0.887
**CD8^+^ Tem cell (cells/μL) (High vs Low)**	57	0.45	0.780
**CD8^+^ Tte cell (cells/μL) (High vs Low)**	15	0.978	1.009

Based on analysis above, the percentage and the AC of CD4^+^ Tn, CD4^+^ Tscm, CD4^+^ Tcm, CD4^+^ Tem, CD4^+^ Tte, CD8^+^ Tn, CD8^+^ Tscm, CD8^+^ Tcm, CD8^+^ Tem and CD8^+^ Tte can be divided into high and low groups according to their cutoff-point (**Table 6**). In 92 patients, 71 (77%) had a high percentage of CD4^+^ Tn (≥ 1.8%), of them 18 (25%) were PR, 31 (44%) were SD, 22 (31%) were PD, the disease control rate (DCR) reached 69%, while 21(23%) had a low percentage of CD4^+^ Tn (< 1.8%), of them 1 (5%) were PR, 3 (14%) were SD, 17 (81%) were PD, the DCR was 19% only. Markedly, the percentage of CD4^+^ Tn showed a positive correlation to the DCR, the higher percentage of CD4^+^ Tn (≥ 1.8%) signified a better outcome of immunotherapy, and the lower percentage of CD4^+^ Tn (< 1.8%) implied poorer curative effect. When the AC of CD4^+^ Tn was higher than or equal to 5.5 cells/μL, the DCR was 71%, and the PD rate was 29%. On the contrary, the PD rate was 77% higher than the DCR (23%) when the AC of CD4^+^ Tn was less than 5.5 cells/μL.

**Table 6:** Evaluation response of Tn/Tm of aNSCLCs received immunotherapy.

Cells	Groups	n	Disease control		Progressive Disease
		92	PR = 19	SD = 34	PD = 39
			n (%)	n (%)	n (%)
**CD4^+^ Tn %**	≥ 1.8%	71	18 (25%)	31 (44%)	22 (31%)
	< 1.8%	21	1 (5%)	3 (14%)	17 (81%)
**CD4^+^ Tn**	≥ 5.5 cells/μL	66	19 (29%)	28 (42%)	19 (29%)
	< 5.5 cells/μL	26	0	6 (23%)	20 (77%)
**CD4^+^ Tscm %**	≥ 17%	48	10 (21%)	19 (39.5%)	19 (39.5%)
	< 17%	44	9 (20%)	15 (34%)	20 (46%)
**CD4^+^ Tscm**	≥ 64.5 cells/μL	46	14 (30%)	21 (46%)	11 (24%)
	< 64.5 cells/μL	46	5 (11%)	13 (28%)	28 (61%)
**CD4^+^ Tcm %**	≥ 29.85%	46	12 (26%)	19 (41%)	15 (33%)
	< 29.85%	46	7 (15%)	15 (33%)	24 (52%)
**CD4^+^ Tcm**	≥ 116.5 cells/μL	46	14 (30%)	21 (46%)	11 (24%)
	< 116.5 cells/μL	46	5 (11%)	13 (28%)	28 (61%)
**CD4^+^ Tem %**	≥ 22.1%	47	11 (23%)	19 (41%)	17 (36%)
	< 22.1%	45	8 (18%)	15 (33%)	22 (49%)
**CD4^+^ Tem**	≥ 80 cells/μL	46	14 (30%)	21 (46%)	11 (24%)
	< 80 cells/μL	46	5 (11%)	13 (28%)	28 (61%)
**CD4^+^ Tte %**	≥ 0.35%	56	11 (20%)	21 (38%)	24 (42%)
	< 0.35%	36	8 (22%)	13 (36%)	15 (42%)
**CD4^+^ Tte**	≥ 2 cells/μL	50	11 (22%)	19 (38%)	20 (40%)
	< 2 cells/μL	42	8 (19%)	15 (36%)	19 (45%)
**CD8^+^ Tn %**	≥ 1.35%	30	5 (17%)	16 (53%)	9 (30%)
	< 1.35%	62	14 (23%)	18 (29%)	30 (48%)
**CD8^+^ Tn**	≥ 2 cells/μL	59	11 (19%)	30 (51%)	18 (30%)
	< 2 cells/μL	33	8 (24%)	4 (12%)	21 (64%)
**CD8^+^ Tscm %**	≥ 9.55%	44	8 (18%)	19 (43%)	17 (39%)
	< 9.55%	48	11 (23%)	15 (31%)	22 (46%)
**CD8^+^ Tscm**	≥ 23.5 cells/μL	43	11 (26%)	23 (53%)	9 (21%)
	< 23.5 cells/μL	49	8 (16%)	11 (23%)	30 (61%)
**CD8^+^ Tcm %**	≥ 9%	47	10 (21%)	18 (38%)	19 (41%)
	< 9%	45	9 (20%)	16 (36%)	20 (44%)
**CD8^+^ Tcm**	≥ 25.5 cells/μL	46	12 (26%)	19 (41%)	15 (33%)
	< 25.5 cells/μL	46	7 (15%)	15 (33%)	24 (52%)
**CD8^+^ Tem %**	≥ 21.3%	46	10 (22%)	15 (33%)	21 (45%)
	< 21.3%	46	9 (20%)	19 (41%)	18 (39%)
**CD8^+^ Tem**	≥ 57 cells/μL	47	12 (26%)	17 (36%)	18 (38%)
	< 57 cells/μL	45	7 (15%)	17 (38%)	21 (47%)
**CD8^+^ Tte %**	≥ 6.55%	46	9 (20%)	20 (44%)	17 (36%)
	< 6.55%	46	10 (22%)	14 (30%)	22 (48%)
**CD8^+^ Tte**	≥ 15 cells/μL	48	11 (23%)	23 (48%)	14 (29%)
	< 15 cells/μL	44	8 (18%)	11 (25%)	25 (57%)

The AC of Tscm, Tcm, and Tem of CD4^+^ as well as Tn, Tscm, Tcm, and Tte of CD8^+^ all showed similar characteristics to the AC and percentage of CD4^+^ Tn. The same applies to the percentage of CD4^+^ Tcm (**Table 6**). The higher AC (≥ cutoff-point) had better DCR than the lower AC (< cutoff-point). In other words, the lower AC had a higher PD rate.

Due to a small fraction of higher percentage and AC (≥ cutoff-point) of Tn/Tm showing no better efficacy (**Table 6**), to explore the cause, we further analyzed the correlation between response of immunotherapy regimes and the AC of CD4^+^ Tn (**Table 7**). Here is an analysis relationship between the AC of CD4^+^ Tn and immunotherapy only. The reason was that multivariate analysis above showed that the AC of CD4^+^ Tn was the most significant protective factor for PFS of patients (**Figures 6**, **8**). For high AC of CD4^+^ Tn (≥ 5.5 cells/μL) (n = 66), immunotherapy alone achieved 3 (16%) PR, 5 (18%) SD, 5 (26%) PD; for low AC of CD4^+^ Tn cell (< 5.5 cells/μL) (n = 26), gained 0 PR, 1 (17%) SD and 4 (20%) PD. Compared to it, immunotherapy conjugate Astragalus polysaccharide (APS) showed better efficacy with PR (5, 26%) and SD (8, 28.5%) increase, PD (3, 16%) drop for high AC of CD4^+^ Tn (≥ 5.5 cells/μL), meanwhile PR (0), SD (1, 17%) and PD (1, 5%) decrease for low AC of CD4^+^ Tn (< 5.5 cells/μL). Immunotherapy followed by chemotherapy showed more better efficacy with PR (7, 37%), SD (9, 31.5%) increase, PD (4, 21%) decrease for high AC of CD4^+^ Tn (≥ 5.5 cells/μL), and SD (2, 33%) increase and PD (4, 20%) steadiness for low AC of CD4^+^ Tn (< 5.5 cells/μL). Immunotherapy conjugate recombinant human Granulocyte Colony Stimulating Factor (G-CSF) didn’t display better outcomes with PR, SD, and PD drop for high AC of CD4^+^ Tn (≥ 5.5 cells/μL), and SD and PD decreased too for low AC of CD4^+^ Tn (< 5.5 cells/μL). Chemotherapy followed by immunotherapy revealed poorer efficacy with PR and SD drop, PD rise for high AC of CD4^+^ Tn cell (≥ 5.5 cells/μL), simultaneously, PD increase apparently, for low AC of CD4^+^ Tn cell (< 5.5 cells/μL) (**Table 7**).

**Table 7:** Frequency for response of treatment options in the AC of CD4^+^ Tn cell in aNSCLCs.

Treatment regime	CD4^+^ Tn cell					
	≥ 5.5 cells/μL (n = 66)			< 5.5 cells/μL (n = 26)		
	PR	SD	PD	PR	SD	PD
**n**	19	28	19	0	6	20
Immunotherapy alone	3 (16%)	5 (18%)	5(26%)	0	1(17%)	4 (20%)
Immunotherapy conjugate APS	5 (26%)	8 (28.5%)	3 (16%)	0	1 (17%)	1 (5%)
Immunotherapy conjugate Recombinant Human G-CSF	2 (10.5%)	3 (11%)	1 (5%)	0	0	1 (5%)
Chemotherapy followed by immunotherapy	2 (10.5%)	3 (11%)	6 (32%)	0	2 (33%)	10 (50%)
Immunotherapy followed by chemotherapy	7 (37%)	9 (31.5%)	4 (21%)	0	2 (33%)	4 (20%)

APS, Astragalus polysaccharide; G-CSF, Granulocyte Colony Stimulating Factor.

The results indicated the efficacy of immunotherapy conjugate chemotherapy was related to the AC of CD4^+^ Tn and treatment order. The high AC of CD4^+^ Tn, and chemotherapy used after immunotherapy predicted a better prognosis, and it was recommended options for the clinic.

## Discussion

The T lymphocytes play a crucial role in protecting the body against cancer by recognizing and killing tumor cells (39, 40). T lymphocytes have mainly divided into naïve T cells (Tn) and memory T cells (Tm) based on their function and phenotype (41). Tn is a mature T cell that is resting. Tn performs the immune-surveillance function by recirculating between the blood and secondary lymphoid organs, and Tn responds only to new antigens (42). DC capture tumor antigens and present them through HLA class II or I molecules to CD4^+^ Tn or CD8^+^ Tn that can differentiate into Th and memory CD4^+^ T cells or memory CD8^+^ T cells, also known as the initial immune response (43). After 90-95% of effector T cells undergo apoptosis, only 5-10% of activated T cells were converted into long-term Tm. These Tm persisted and were involved in maintaining long-term immunity and rapid responses to antigens that had been exposed, also known as memory immune responses (44, 45). Tn and Tscm could self-renew and differentiate into all subsets of memory and effector T cells, which include Tcm, Tem, and Tte (46, 47). Tscm and Tcm undergo a memory immune response and rapidly clonally proliferate to produce Tem and Tte that specifically kill tumor cells (48). Our study showed the AC of Tn/Tn impaired significantly in aNSCLCs, the insufficient numbers of Tn or Tscm and Tcm cannot induce enough initial immune responses and memory immune responses, and cannot recognize and kill tumor cells, which may explain the development of refractory tumors. Hence, different T lymphocytes coordinate with each other and play a crucial role in the anti-tumor immune response (49, 50). Therefore, studying the number and function of Tn and Tm cells is a reference for understanding anti-tumor immunity.

Most studies have focused on the frequency of immune cells in blood or tumor-infiltrating lymphocytes (TILs) and served as biomarkers of prognosis (51–53). Liu C, et al. reported that the percentage of CD4^+^ Tn and Tm in peripheral blood are prognostic indicators for patients with NSCLC (54–56). However, the “Tn” of these researches was a mixture of Tn and Tscm due to not using CD95 monoclonal antibodies to discriminate Tscm from Tn according to the methods of the papers (57), while Tn and Tscm in our research had distinguished by CD95 monoclonal antibody. The “Tn” in these researches was completely different from the “Tn” of our manuscript. Meanwhile, this researches only analyzed the memory CD4^+^ and CD8^+^ T cells but did not fully analyze the subsets of memory cells, including Tscm, Tcm, Tem, and Tte (30, 54–56). More importantly, these studies did not analyze further the prognostic impact of each AC of the Tn/Tm subpopulation on patient survival. Therefore, naïve and memory cells of CD4^+^ and CD8^+^ T cells in the research are completely different from our manuscript. Wherever, the impact of AC in the Tn/Tm subpopulation of peripheral blood, which represents the overall immune status, on the prognosis of NSCLC is unclear. Chen DS, et al. indicated that possible reasons for the absence of the T-cell infiltration in the tumor microenvironment (TME) include lack of tumor antigens, defects in antigen-presenting cells (APCs), and lack of T-cell activation (58). Yost KE, et al. reported that the T-cell clones that dominate the T-cell landscape within the tumor after ICB are distinct from that before treatment, a phenomenon referred to by the authors as a clonal substitution (59). More and more evidence indicate that cloning and recruitment of T lymphocytes may be a key to disease progression during immunotherapy (60). It had suggested that the reactivation capacity of TILs was limited, and the type of expansion of T cell clones after immunotherapy had recruited from peripheral sources (59). Deep single-cell sequencing of RNA and T cell receptors in the patient’s peripheral blood, tumor samples, and normal adjacent tissues confirmed the source of clonotypic amplification of TILs. The results indicated that in patients who responded, TILs were replaced by fresh non-depleted T cells from outside the tumor (61). Therefore, TILs in the local TME may be derived from Tn/Tm subsets in the peripheral blood. The immune status of the patient’s TME is crucial in terms of their prognosis. We speculate that the TME combined with the systemic immune status of patients is more relevant for the prognosis of patients and the stratification of patients for immunotherapy. We hypothesized that adequate numbers of Tn/Tm are considerable for maintaining the immune homeostasis in the local TME. It is necessary to determine which AC of Tn/Tm in peripheral blood have closely associated with the prognosis of the tumor. Our previous study showed that AC of CD4^+^ cells had a predictive value in longer PFS of NSCLC, while the percentages of CD3^+^, CD4^+^, CD8^+^, B, and NK cells were the same as that in NCs, with no difference between the two groups (36). The frequency and AC of T cells reflect two aspects of immune cells: the frequency reflects mainly the development and differentiation of immune cells, while the AC shows the proliferation of immune cells. The state of immune cells requires analysis of both the percentage and AC of cells (62). Studies have shown that the AC of Tn is lower in patients with NSCLC than that in normal subjects (63).

Notably, we focused on Tn/Tm in this study. The percentage and AC of Tn/Tm had severely reduced in aNSCLCs compared to NCs, and the decrease in AC was more regular, especially the AC of CD4^+^ Tn and CD8^+^ Tn was associated with the disease stage of the tumor, that is, with the disease progression of aNSCLCs (**Figure 3**). This result suggested that aNSCLCs lack sufficient numbers of Tn to generate initial immune responses by DC recognizing and intaking new tumor antigens. This condition might cause the recurrence and metastasis of tumor cells (**Figure 4**). The data indicated that the initial immune response of Tn and proliferation of the T cell in aNSCLCs were severely impaired. And the inability to effectively perform immune surveillance and clearance might lead to the metastasis and progression of NSCLC.

Some studies have shown that immunotherapy conjugate with radiotherapy did not improve the outcome of aNSCLCs (21). We inferred that the ineffectiveness of immunotherapy could be due to the depletion of CD4^+^ Tn, leading directly to desensitization to new antigens produced by recurrent tumors, which coincides with the significant impairment in AC of Tn in aNSCLCs. Therefore, it may be a crucial factor in the formation of clinically refractory tumors (**Figure 6**).

Multivariate analysis of all variables of Tn/Tm related to the prognosis of aNSCLCs showed that the AC of CD4^+^ Tn (≥ cutoff-point) in peripheral blood was an independent protective factor in PFS of aNSCLCs (**Figures 6**, **8**). Immunotherapy acts by indirectly relieving immune suppression of immune cells rather than directly killing tumor cells. These results indicated that the AC of CD4+ Tn is not only a prognostic indicator for aNSCLC but also strongly correlates with the efficacy of immunotherapy. More importantly, the population benefiting from immunotherapy can be screened out from aNSCLCs based on the AC of CD4^+^ Tn. It is significant to improve efficacy and reduce ineffective immunotherapy in the clinic.

Studies have shown that immunotherapy can promote the differentiation of Th cells and activation of CTLs in responded patients, but that didn’t happen with chemotherapy (64). Analysis of T lymphocyte subsets of peripheral blood in patients with metastatic melanoma after initial immunotherapy showed increased expansion of peripheral T lymphocyte subsets in patients who responded to treatment (65). Considering that immunotherapy strategies are also important factors, we further explored the relationship between the treatment regime and the AC of CD4^+^ Tn.

For aNSCLC, adequate Tn is a prerequisite for immunotherapy to be effective. Immunotherapy combined with other immune-enhancing therapies can further improve outcomes, such as APS, which can significantly improve the function of humoral immunity and cellular metabolism (66, 67). Chemotherapy can shrink tumors that are sensitive to cytotoxic drugs. However, chemotherapy can cause side effects, such as bone marrow suppression (68–70). In particular, repeated chemotherapy can impair the proliferative function of cells, including the rapidly proliferating hematopoietic stem cells in the bone marrow and Tn, Tscm, and Tcm in the peripheral blood. As a result, immune dysfunction occurs when immune cells do not complete the initial and memory immune responses performed by Tn and Tm. When CD4^+^ Tn ≥ 5.5 cells/μL, immunotherapy followed by chemotherapy had the highest DCR in our study. Immunotherapy alone also had a higher DCR than chemotherapy followed by immunotherapy. Therefore, the sequence of treatment regimens could affect the prognosis and efficacy of patients. Chemotherapy should be avoided in patients when the AC of Tn is low, which reduces damage to immune cells, and immune-boosting therapy should be used at this time. Different clinical strategies should be used for patients at different clinical stages, depending on the patient’s immune status. In brief, these results suggest that the efficacy of immunotherapy is closely related to the AC of CD4^+^ Tn.

This study analyzed the impaired Tn/Tm in aNSCLCs, its prognostic value for immunotherapy, and implications for combination treatment strategies. However, the study has some limitations. For example, it lacks further clinical validation in large samples, a multi-center study of CD4^+^ Tn as a biomarker, and the mechanisms of Tn/Tm impairment.

## Conclusion

This study suggested that the percentage and AC of Tn/Tm, especially AC, showed varying degrees of damage in aNSCLC and were associated with tumor progression. The multivariate analysis indicated that only AC of CD4^+^ Tn was an independent protective factor for PFS in aNSCLCs and a potential marker of efficacy and prognosis in patients receiving immunotherapy. The AC of Tn/Tm test in peripheral blood is simple to perform, cost saving and helps to assess the overall immune status of aNSCLC patients for clinical management. Moreover, our finding suggests that immunotherapy conjugate other therapies such as APS or followed by chemotherapy is of benefit for aNSCLCs.

## Data availability statement

The original contributions presented in the study are included in the article/**Supplementary Material**. Further inquiries can be directed to the corresponding author.

## Ethics statement

All enrolled participants signed the informed consent. The present study involving human samples was approved by the Ethics Committee of the First Teaching Hospital, Tianjin University of Traditional Chinese Medicine (Tianjin, China) (TYLL2021 [K] 001).

## Author contributions

GZ drafted the manuscript. JY conceived and designed manuscript. AL, YY, YX, WL, YL and JZ verified the contents and revised the manuscript. QC, DW, XL, YG, HC critically revised the manuscript. All authors reviewed and approved the final manuscript. All authors contributed to the article and approved the submitted version.

## Funding

This work was supported by the Scientific Research Plan Project of Tianjin Education Commission (No. 2018KJ034).

## Acknowledgments

The authors thank the Scientific Research Plan Project of Tianjin Education Commission (No.2018KJ034).

## Conflict of interest

The authors declare that the research was conducted in the absence of any commercial or financial relationships that could be construed as a potential conflict of interest.

## Publisher’s note

All claims expressed in this article are solely those of the authors and do not necessarily represent those of their affiliated organizations, or those of the publisher, the editors and the reviewers. Any product that may be evaluated in this article, or claim that may be made by its manufacturer, is not guaranteed or endorsed by the publisher.

## Supplementary material

Supplementary Figure 1 Prognostic impact of naïve/memory T lymphocytes on PFS of NSCLCs: **(A)** The percentage of CD4^+^ Tcm on the prognosis. **(B)** The AC of CD4^+^ Tcm on the prognosis. **(C)** The AC of CD4^+^ Tem on the prognosis. **(D)** The percentage of CD4^+^ Tte on the prognosis. **(E)** The AC of CD4^+^ Tte on the prognosis. **(F)** The AC of CD8^+^ Tscm on the prognosis. **(G)** The percentage of CD8^+^ Tcm on the prognosis. **(H)** The AC of CD8^+^ Tcm on the prognosis. **(I)** The percentage of CD8^+^ Tem on the prognosis. **(J)** The AC of CD8^+^ Tem on the prognosis. **(K)** The percentage of CD8^+^ Tte on the prognosis. **(L)** The AC of CD8^+^ Tte on the prognosis.

Supplementary Figure 2 Prognostic impact of naïve/memory T lymphocytes on PFS of NSCLCs received immunotherapy: **(A)** The percentage of CD8^+^ Tscm on the prognosis. **(B)** The percentage of CD4^+^ Tcm on the prognosis. **(C)** The AC of CD4^+^ Tcm on the prognosis. **(D)** The percentage of CD4^+^ Tem on the prognosis. **(E)**The AC of CD4^+^ Tem on the prognosis. **(F)** The percentage of CD4^+^ Tte on the prognosis. **(G)** The AC of CD4^+^ Tte on the prognosis. **(H)** The percentage of CD8^+^ Tn on the prognosis. **(I)** The AC of CD8^+^ Tn on the prognosis. **(J)** The percentage of CD8^+^ Tcm on the prognosis. **(K)** The AC of CD8^+^ Tcm on the prognosis. **(L)** The percentage of CD8^+^ Tem on the prognosis. **(M)** The AC of CD8^+^ Tem on the prognosis. **(N)** The percentage of CD8^+^ Tte on the prognosis. **(O)** The AC of CD8^+^ Tte on the prognosis.
