# Supplementary figures and images for: Clinical predictive value of naïve and memory T cells in advanced NSCLC
Source: Front Immunol. 2022 Sep 2;13:996348. doi: 10.3389/fimmu.2022.996348 (PMC9478592; doi:10.3389/fimmu.2022.996348)

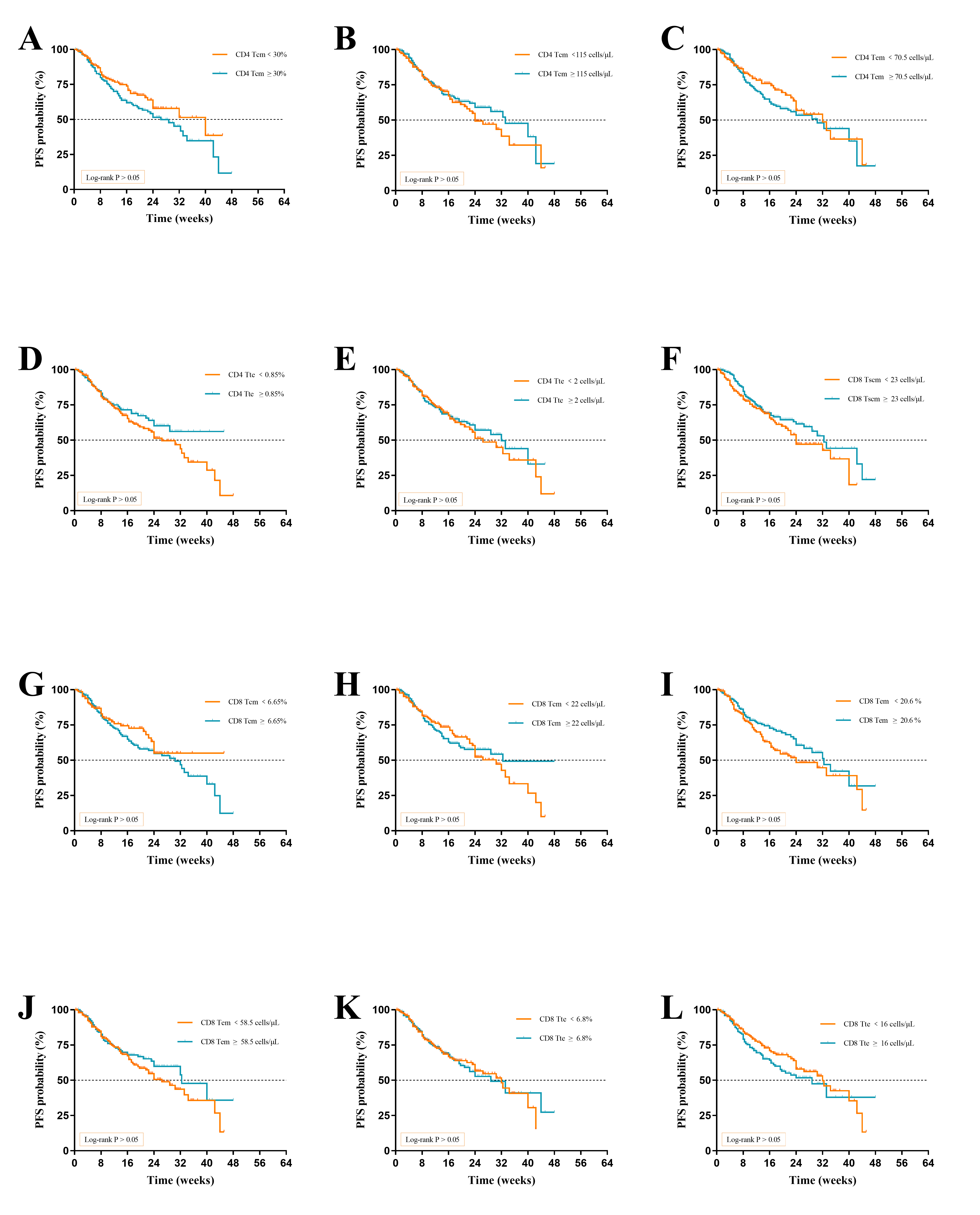

Supplement: Supplementary Figure 1 — Prognostic impact of naïve/memory T lymphocytes on PFS of NSCLCs: (A) The percentage of CD4+ Tcm on the prognosis. (B) The AC of CD4+ Tcm on the prognosis. (C) The AC of CD4+ Tem on the prognosis. (D) The percentage of CD4+ Tte on the prognosis. (E) The AC of CD4+ Tte on the prognosis. (F) The AC of CD8+ Tscm on the prognosis. (G) The percentage of CD8+ Tcm on the prognosis. (H) The AC of CD8+ Tcm on the prognosis. (I) The percentage of CD8+ Tem on the prognosis. (J) The AC of CD8+ Tem on the prognosis. (K) The percentage of CD8+ Tte on the prognosis. (L) The AC of CD8+ Tte on the prognosis. [file Image_1.tif]

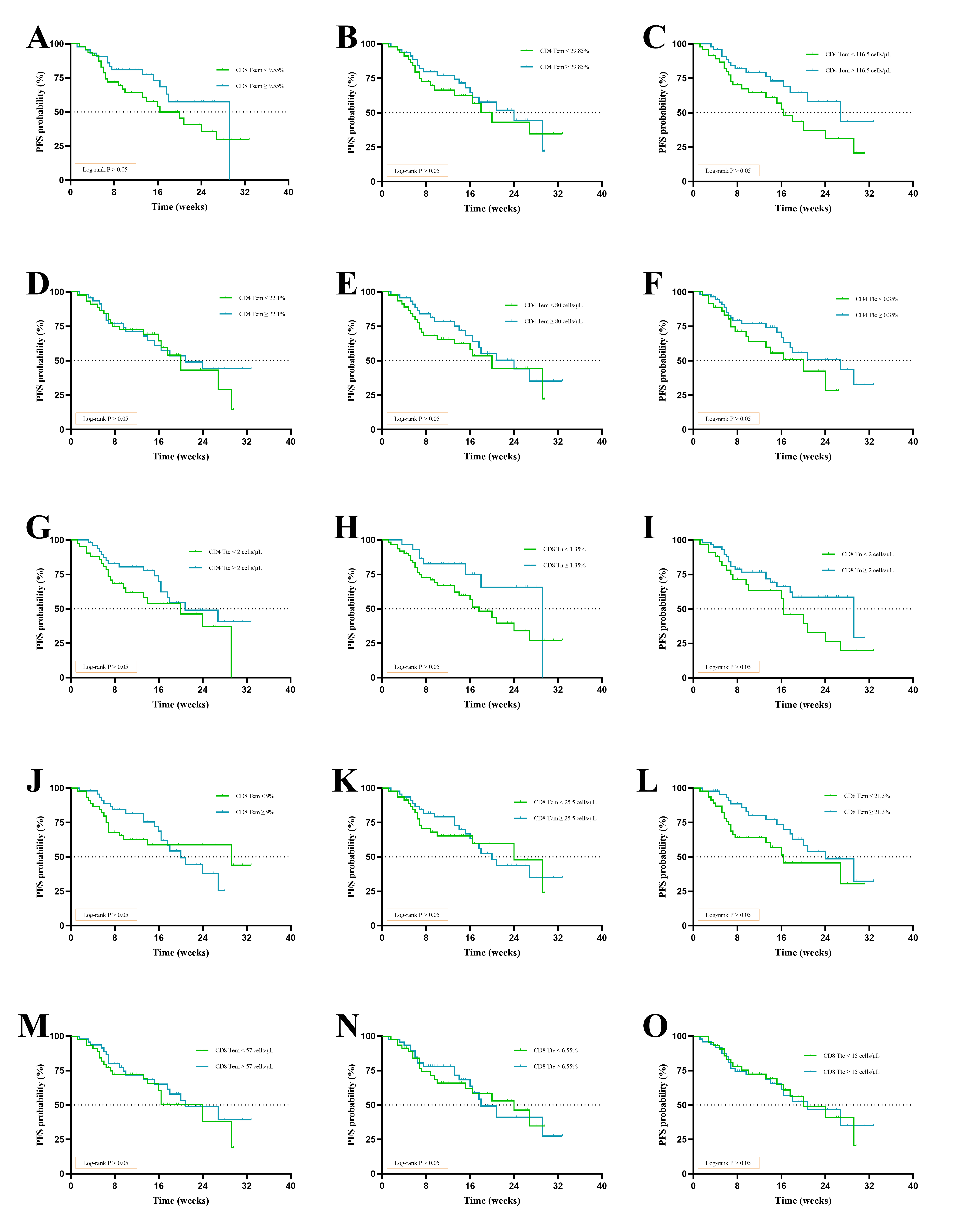

Supplement: Supplementary Figure 2 — Prognostic impact of naïve/memory T lymphocytes on PFS of NSCLCs received immunotherapy: (A) The percentage of CD8+ Tscm on the prognosis. (B) The percentage of CD4+ Tcm on the prognosis. (C) The AC of CD4+ Tcm on the prognosis. (D) The percentage of CD4+ Tem on the prognosis. (E)The AC of CD4+ Tem on the prognosis. (F) The percentage of CD4+ Tte on the prognosis. (G) The AC of CD4+ Tte on the prognosis. (H) The percentage of CD8+ Tn on the prognosis. (I) The AC of CD8+ Tn on the prognosis. (J) The percentage of CD8+ Tcm on the prognosis. (K) The AC of CD8+ Tcm on the prognosis. (L) The percentage of CD8+ Tem on the prognosis. (M) The AC of CD8+ Tem on the prognosis. (N) The percentage of CD8+ Tte on the prognosis. (O) The AC of CD8+ Tte on the prognosis. [file Image_2.tif]
